# Variation in the 11-year trajectories of medical care seeking behaviors in diabetes patients under a single payer system: persisting gaps to be filled

**DOI:** 10.1186/s12913-019-4399-0

**Published:** 2019-08-19

**Authors:** Tzu-Ho Tsai, Nicole Huang, I-Feng Lin, Yiing-Jenq Chou

**Affiliations:** 1 Institute of Public Health, School of Medicine, National Yang-Ming University, No.115, Sec. 2, Linong Street, Taipei, Taiwan; 2 Department of Intensive Care, Cheng-Hsin Hospital, No. 45, Cheng Hsin Street, Taipei, Taiwan; 3 Institute of Hospital and Health Care Administration, School of Medicine, National Yang-Ming University, No.115, Sec. 2, Linong Street, Taipei, Taiwan

**Keywords:** Healthcare-seeking behaviors, Medical adherence, Healthcare provider, Diabetes mellitus, Trajectory

## Abstract

**Background:**

Care-seeking behavior is widely acknowledged to have strong influences on health outcomes among individuals with chronic conditions including diabetes. Despite its dynamic nature, care seeking behavior are often considered as time invariant in most studies. The likelihood of patients changing their regularity and source of chronic care over time is often neglected. This study aimed to determine the long-term trajectories of care-seeking patterns of both care-seeking regularity and health provider choices; and their associated factors among patients with type 2 diabetes under the National Health Insurance (NHI) program in Taiwan.

**Methods:**

We utilized population-based data from the National Health Insurance Research Database (NHIRD) in Taiwan. Three thousand, nine hundred and eighty-seven adult patients with newly diagnosed type 2 diabetes in 1999 were enrolled in the cohort. We assessed their trajectories of regular care visits and sources of diabetes care from 2000 to 2010. A group-based trajectory model was applied.

**Results:**

Seven distinct groups of long-term care-seeking patterns were identified. Only 51.44% of patients with newly diagnosed diabetes had regularly visited their providers over time. Among them, 56.41 and 16.09% had persistently sought care from generalized and specialized providers, respectively. 27.50% had sought care from different levels of providers. Patients who were male, elderly, low-income, and had a higher baseline diabetes severity were significantly more likely to either continue with their irregular care-seeking behavior or fail to maintain their regular care seeking behavior over time. Those who were younger, had a higher socioeconomic status, and lived in an urban area were significantly more likely to persistently seek care from specialized care settings.

**Conclusions:**

This study is the first population-based assessment of long-term care-seeking behaviors of type 2 diabetes patients under a single-payer system with a comprehensive benefit coverage. The most alarming finding was that, despite the existence of the comprehensive universal health insurance coverage in Taiwan, almost 50% of patients did not seek or maintain regular visits to providers over time as recommended. Understanding variations in the long-term trajectories of care adherence and sources of care may help to identify gaps in diabetes care management.

## Background

Diabetes mellitus is a prevalent and challenging chronic condition, which levies heavy care and financial burdens on individuals, families, providers and societies [1]. Care-seeking behavior is widely acknowledged to have strong influences on health outcomes among individuals with chronic conditions including diabetes [2]. Two aspects of health care-seeking behaviors are particularly critical in the management of diabetes: adherence to regular physician visits and choice of the health care source [3–6]. Diabetes patients often underestimate the importance of regular follow-ups because its complications are typically asymptomatic until the very late stages of the disease. Regular visits to diabetes providers are widely recognized as one most critical strategy in diabetes care management, which may help improving patient outcomes such as HbA1c and reducing preventable hospital admissions [7–9]. Patient adherence to regular health care is influenced by the patients’ demographic characteristics, socioeconomic status (SES), health status, geographical location, and treatment factors [10]. Understanding the factors driving heterogeneity in regular care seeking behaviors among diabetes patients can help to identify population targets and design subpopulation-specific strategies for better patients’ compliance with recommended care.

In addition, patient sources of care have been extensively discussed. Providers with different levels of specialization or qualifications may have different management styles and yield different quality of diabetes care [11, 12]. In addition, in a fragmented health care system with flexible provider choices such as in Taiwan and several East Asian countries, different care settings may yield large differences in costs. Outpatient practices for treating the same conditions in academic medical centers or advanced teaching hospitals tend to be reimbursed at a higher rate and lead to higher charges than non-academic provider settings (i.e. physician offices or local hospital outpatient practices). Identifying and characterizing long-term care seeking behaviors of diabetes patients allows us to gain more knowledge of long-term patient flow across different settings and resource allocation pattern among diabetes patients. Such information helps to indicate potential rooms for improvement in efficiency. A review article reported large variations in the preferred provider size and specialization among patients with chronic conditions [13]. Some studies have also suggested that patients with chronic illness prefer an university medical hospital with multiple specialists [14, 15]. In contrast, another study showed that patients prefer generalized providers [16]. Patient preference of service providers level may be influenced by age, gender, SES, illness type, access to services, and the perceived quality of services [17–19]. Van Doorslaer et al. illustrated significant socioeconomic inequity in visits to general practitioners and specialists in many countries [20]. Elderly patients, female patients, patients with private insurance or who are extensively insured, and patients with chronic conditions and having poor perceived health tended to visit a specialist rather than a general practitioner [21].

Although many studies have investigated the care-seeking behaviors of patients with diabetes, most of these studies have employed a cross-sectional design. Cross-sectional measurement of care seeking behaviors cannot describe the likely time-varying nature of care seeking behaviors of patients with diabetes or other chronic conditions. The likelihood of patients changing their health care providers and regularity of provider visits over time is often neglected. In this study, we tried to address this gap by identifying distinct groups of patients with newly diagnosed type 2 diabetes who exhibited similar trajectories of regular care visits and sources of diabetes care. For care management of chronic conditions like type 2 diabetes, characterizing variation in patients’ longitudinal care seeking patterns is essential to understanding the factors driving heterogeneity in care seeking behaviors, and it can help to identify targets for strategies designed to close gaps in care delivery and improve quality.

Taiwan serves an interesting setting for this study as it has established a single-payer universal health care insurance program since 1995. All Taiwanese residents are enrolled in this National Health Insurance (NHI) program and more than 90% of health care providers are contracted with the NHI program [22]. The NHI program is renowned for its comprehensive benefit coverage and freedom of provider choice. Along with these interesting features, the availability of longitudinal and comprehensive administrative data allowed us to explore the longitudinal care seeking patterns of diabetes patients and identify baseline characteristics associated with trajectories of care seeking behaviors.

## Methods

Since its establishment in March 1995, the NHI program in Taiwan offers comprehensive benefit coverage to all Taiwanese residents. In spite of the mandatory nature of program enrollment, people have a relatively flexible freedom in provider choices [23]. In Taiwan, health care providers are categorized into four levels according to the level of specialization in personnel training and the services offered: medical centers, regional hospitals, district hospitals, and clinics/private physician offices. Most hospitals in Taiwan operate a large outpatient department. No referral is necessary for accessing higher-level providers or specialists. Only a slightly higher copayment is levied on ambulatory care visits to medical centers and regional hospitals without referral. The difference in co-payment between visits to higher-level hospitals with and without referral ranges from 140 NTD (~ 4.7 USD) to 250 NTD (~ 8.3 USD). Such differences do not seem to deter patients from directly visiting medical centers and regional hospitals for common chronic conditions, such as diabetes. No other strict referral restrictions are enforced. Self-referral is common in Taiwan.

This retrospective longitudinal cohort study utilized data from the Longitudinal Health Insurance Database (LHID) 2000. The quality of insurance claims data of patients with diabetes in Taiwan has been validated [24]. The LHID2000 contains the detailed claims data of one million patients randomly sampled from all NHI enrollees in 2000. The LHID2000 includes the following data: (1) The registry for beneficiaries provides patients’ information on date of birth, gender, insurable income, status and types of enrollment. (2) The registry for contracted medical facilities provide information on the NHI-contracted medical institutions. The level and location of health care facilities are recorded. (3) The claims databases provide information on the date of each visit, diagnosis information in International Classification of Diseases, Ninth Revision, Clinical Modification (ICD-9-CM) codes, types of health providers, and types and costs of all health care services.

**Fig. 1:**
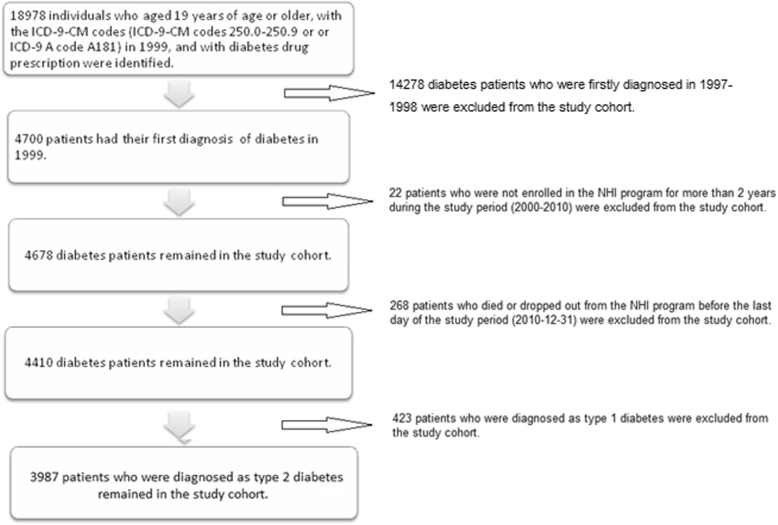
Flow chart for selection of study participants

We firstly identified adult patients with newly diagnosed diabetes as those aged 19 years of age or older and had their first diagnosis of diabetes in 1999. We included 18,978 diabetes patients with ICD-9-CM codes 250.0–250.9 or ICD-9 A code A181 [25]. To ensure the patients identified were incident diabetes cases, those with any diagnosis of diabetes reported in prior years (1997–1998) were excluded. To avoid potential rule-out diagnosis, we only included 4700 patients who had not only a diagnosis of diabetes, but also were prescribed diabetes drugs. Two hundred and ninety diabetes patients who were not covered under the NHI program for more than 2 years during 2000 to 2010 and those who died or dropped out of the NHI program before and at December 31, 2010 were excluded from the study. Four hundred and twenty-three type 1 diabetes patients were also excluded from the study by using ICD-9-CM codes of type 1 DM such as 250.01, 250.11, 250.13, 250.41, 250.43, 250.51, 250.53, 250.61, 250.63, 250.71, 250.73, 250.91, and 250.93 (https://www.medicalhomeportal.org/link/3917). The final sample included 3987 adult patients with newly diagnosed type 2 diabetes in 1999. Figure 1 shows the flowchart of the process mentioned above. We assessed the trajectories of their care-seeking patterns from January 1, 2000, to December 31, 2010.

This study assessed two aspects of care-seeking behaviors: regular visits to providers and level of providers sought. According to the guideline for diabetes care, patients with diabetes, particularly those patients who are already on medication, should be regularly followed and should undergo hemoglobin A1c assessment at least once every 3 months [26, 27]. Hence, we defined regular care seeking for patients with diabetes as an interval of less than 90 days between each diabetes visit, and more than three visits in each person-year. Regarding the specialization level of the providers, in Taiwan, medical centers and regional hospitals are typically perceived as being more specialized because they need to be equipped with more updated medical technologies and maintain a higher scope of services and personnel as well as higher accreditation standard than district hospitals and clinics do. Hence, we aggregated medical centers and regional hospitals as specialized providers, and district hospitals and clinics as generalized providers. In order to analyze both aspects simultaneously, we combined the two care-seeking aspects into one outcome variable: irregular provider contact, regular contact with providers at more specialized care settings (i.e. medical centers and regional hospitals), and regular contact with providers at generalized care settings (district hospitals and clinics/private physician offices). Outpatient visits for diabetes care were identified as visits with a ICD-9-CM code for diabetes mellitus. To exclude physician visits not primarily for diabetes care, we excluded visits to orthopedic surgeons, otolaryngologist, dentists, radiologists, anesthesiologists, and pathologists. The highest level of provider setting visited by the patients in 1 year was considered the type of provider sought by them in that year. Patients who did not adhere to the guideline (i.e. < 4 diabetes-related visits per year) in one person-year were categorized as 1. Patients with regular visits to providers at generalized care settings were categorized as 2. Patients with regular visits to providers at specialized care settings were categorized as 3.

Sensitivity analysis were performed by including patients with diabetes who were excluded from the study cohort, by changing the interval of regular visits for diabetes patients from 90 days to 120 days, and by changing the type of provider sought from highest level of provider setting visited to the most frequent level of provider setting visited by patients in the observed year. Furthermore, we carried the sensitivity analyses by including patients who died during the study period. These results, such as the distribution and curves of trajectories, remained unchanged.

Predictors of group membership (i.e. distinct trajectory), namely gender, age, socioeconomic status (SES), residential location in 2000, and baseline severity of new-onset type 2 diabetes, were assessed. The SES of patients was defined according to their income categorization for insurance purposes. The NHI program is financed by payroll taxes on people with a well-defined monthly income and head taxes on people without a well-defined monthly income. People with a well-defined monthly income are classified into three categories: ≥NTD40,000, NTD20,000-NTD39,999, or < NTD20,000. People without a well-defined monthly income can enroll in the NHI program either through associations or local government offices. People, such as farmers and fishermen, are enrolled through occupation-related associations. Unemployed or low-income people are mostly enrolled through local government offices. The residential location of patients with diabetes was classified into three categories: rural, sub-urban, and urban areas. Following the recent literatures, we adopted the diabetes complications severity index (DCSI) to measure disease severity of type 2 diabetes patients [28]. DCSI includes seven complication categories: retinopathy, nephropathy, neuropathy, cerebrovascular complications, cardiovascular complications, peripheral vascular disease, and metabolic complications [29]. Complications were identified using ICD-9-CM codes from outpatient or inpatient records. The DCSI is the sum of scores of the seven complication categories. The baseline severity of the newly diagnosed patients was measured at 2000 and was constructed as a nominal variable, namely high (DCSI≥2), moderate (DCSI = 1),and low (DCSI = 0) severity levels.

The group-based trajectory model developed by Nagin was applied to identify subgroups of patients with similar longitudinal care seeking patterns based on the outcome variable described in the previous subsection [30–34]. The trajectories of care-seeking patterns were specified using the censored normal distribution model as a part of the Proc TRAJ modeling process for semi-parametric group modeling [32]. The Bayesian information criterion (BIC) value was used to compare model fits across models that include trajectories of various shapes. After identifying the ideal number of groups and shapes, we determined the model adequacy by using average posterior probability (APP) of group membership. An APP of more than 70% indicates that trajectories are well-assigned to their groups [30]. Multinomial logistic regression was used to quantify the multivariable associations between group membership and baseline patient characteristics. As the NHI program strongly recommend patients with common chronic conditions such as diabetes maintaining regular visits to providers and preferably providers at more general care setting for cost and efficiency concerns, the group of patients who had been regularly followed up and persistently sought care from general providers were used as the reference group in the model. Statistical analyses were conducted using the SAS 9.4 statistical software package.

## Results

**Fig. 2:**
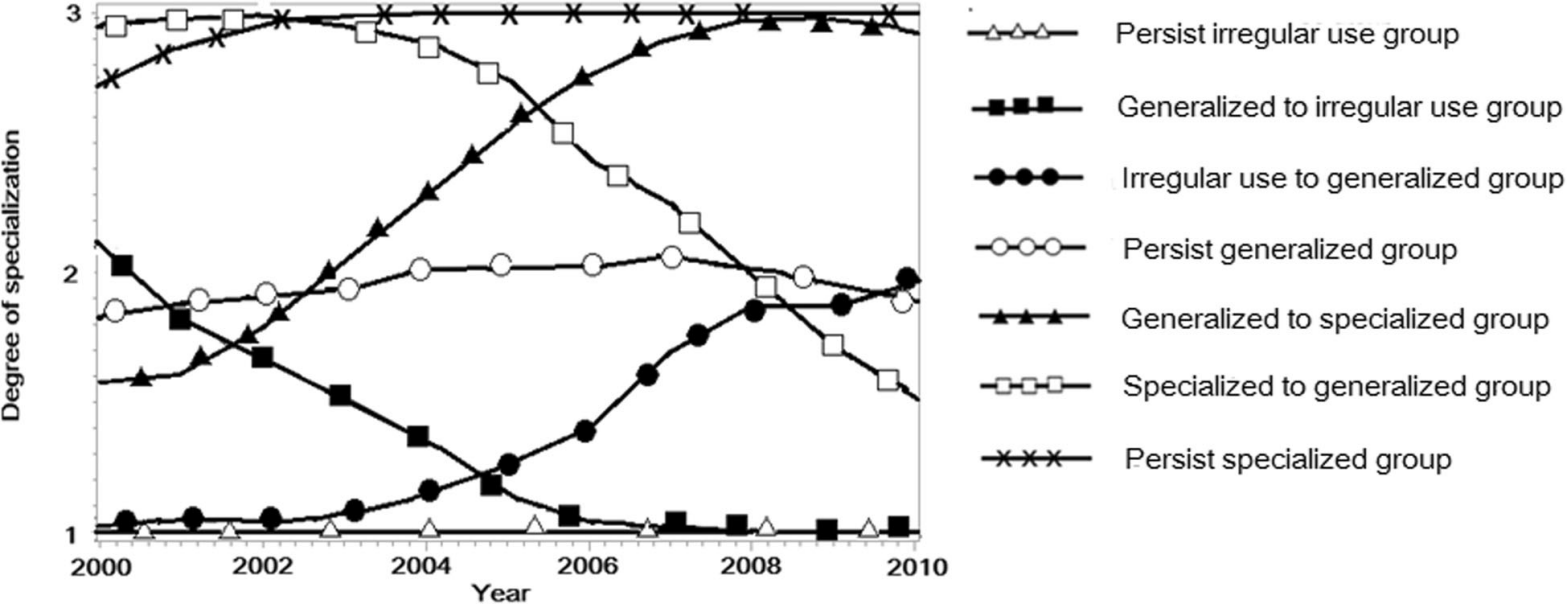
Trajectories of care seeking patterns of patients with newly diagnosed type 2 diabetes from 2000 to 2010. The seven trajectories of health care-seeking behaviors among patients with type 2 diabetes were identified after onset of diabetes from 2000 to 2010. △, white triangles, persist irregular use group (*n* = 1044; 26.19%); ■, black squares, generalized to irregular use group (*n* = 532; 13.34%); ●, black circles, irregular use to generalized group (*n* = 360; 9.03%); ○, white circles, persist generalized group (*n* = 1157; 29.02%); ▲, black triangles, generalized to specialized group (*n* = 351; 8.80%); □, white squares, specialized to generalized group (*n* = 213; 5.34%); and X, crosses, persist specialized group (*n* = 330; 8.28%)

Group-based trajectory models were developed to identify the long-term patterns of care-seeking behaviors among 3987 newly diagnosed type 2 diabetes patients from 2000 to 2010. In all models, we used second-order polynomials for modeling adherence over time. The choice was dependent on repeated observations, and a second-order model was sufficiently flexible to capture relevant changes in care-seeking patterns. We tested models with three to nine trajectories; the BIC values of these models were − 40,910.38, − 39,601.11, − 39,584.52, − 36,973.50, − 36,001.63, − 36,956.92 and − 38,281.96. The seven-group trajectory model most favorably fitted the long-term care-seeking patterns (Fig. 2). The APPs for the seven trajectories ranged from 84.36 to 98.52%, indicating that the trajectories under this model match with their assigned groups. This model identified seven distinct trajectories of long-term care-seeking patterns of patients with newly diagnosed type 2 diabetes (Fig. 2): (1) being persistently irregular users of diabetes care (26.19% of the study population); (2) shifting from regular users of general providers to irregular users (13.34%); (3) shifting from irregular users to regular users of general providers (9.03%); (4) persistently seeking care from general providers (29.02%); (5) shifting from regular users of general providers to that of specialized providers (8.80%); (6) shifting from regular users of specialized providers to that of general providers (5.34%); and (7) persistently seeking care from specialized providers (8.28%).

It is strikingly to find that 26.19% of the patients had persistently failed to maintain regular contacts to any care providers for the entire 11 year period since their first diagnosis of type 2 diabetes. In addition, 13.34% patients who initially had visited generalized providers regularly failed to maintain this regularity over time. Only 9.03% of irregular users gradually retained generalized providers as their regular source of diabetes care over time.

Only 51.44% of patients with newly diagnosed type 2 diabetes in Taiwan persistently and regularly visited their providers during the entire study period from 2000 to 2010. Among them, 56.41% of patients had persistently sought care from generalized providers, 16.09% patients were persistently managed by specialized providers, 17.11% changed their source of diabetes care from a generalized provider to a specialized provider, and 10.39% changed their care source from a specialized to a generalized provider.

Among patients who visited generalized providers initially, 56.72% retained generalized providers as their main source of diabetes care until the end of the 11 years, 17.21% changed their care source to specialized providers, and 26.08% stopped maintaining regular contacts with their providers. Among patients who visited specialized providers at the beginning, 60.77% retained specialized providers as their main source of diabetes care until the end of the trajectories, and 39.23% changed their care source to generalized providers.

Table 1 presents baseline characteristics of patients with newly diagnosed diabetes in Taiwan by group membership. Patients in different trajectory groups differed significantly in their age, SES, and residential location. Table 2 presents adjusted odds ratios (ORs) for predictors of group membership of trajectories. Men were significantly more likely to shift from regular patients at generalized providers to irregular users than remained to be with generalized providers for the whole time (adjusted OR = 1.52, 95% confidence interval [CI]: 1.22,1.88). Furthermore, the older the patients, the more likely they were to lose regular contacts with their providers at generalized settings. Patients aged 75 years or more showed the highest risk of persistently failing to maintain regular contacts with providers (adjusted OR = 3.34, 95%CI: 2.44,4.57) or gradually losing their regular contacts with generalized providers over time (adjusted OR = 5.19, 95%CI: 3.55,7.57). Patients with higher severity were more likely to fail to visit their providers regularly for the whole time (adjusted OR = 1.99, 95%CI: 1.36,2.89) or to gradually lose their regular contacts with generalized providers over time (adjusted OR = 2.26, 95%CI: 1.48,3.44). Patients with moderate severity were the opposite. Considering SES, patients with a monthly income of ≥NTD40,000 (adjusted OR = 1.75, 95%CI: 1.17,2.61) and those who were enrolled through local government agencies (adjusted OR = 1.88, 95%CI: 1.44,2.46) were less likely to maintain a persistent regular visit pattern to providers for diabetes care. Patients who were enrolled through local government agencies were also more likely to gradually lose their regular contacts with their providers at generalized settings (adjusted OR = 1.41, 95%CI: 1.02,1.93).

**Table 1:** Baseline characteristics of diabetes patients stratified by trajectory groups

	Persist irregular use	Generalized to irregular use	Irregular use to generalized	Persist generalized	Generalized to specialized	Specialized to generalized	Persist specialized	*P* ^*^
n (%) Male gender (%)	1044 (26.19) 555 (53.16)	532 (13.34) 313 (58.95)	360 (9.03) 205 (56.94)	1157 (29.02) 565 (48.83)	351 (8.80) 179 (51.00)	213 (5.34) 115 (53.99)	330 (8.28) 166 (50.30)	0.0686
Mean age (years, SD)	61.11 (16.43)	64.03 (13.70)	55.23 (12.60)	57.48 (11.65)	55.03 (10.85)	61.54 (12.24)	57.56 (10.65)	< 0.0001
Age (years, %)								
20–49	278 (26.63)	93 (17.51)	138 (38.33)	320 (27.66)	112 (31.91)	37 (17.37)	74 (22.42)	< 0.0001
50–64	272 (26.05)	146 (27.50)	124 (34.44)	493 (42.61)	162 (46.15)	79 (37.09)	161 (48.79)	
65–74	250 (23.95)	165 (31.07)	77 (21.39)	265 (22.90)	70 (19.94)	63 (29.58)	81 (24.55)	
> =75	244 (23.37)	127 (23.92)	21 (5.83)	79 (6.83)	7 (1.99)	34 (15.96)	14 (4.24)	
Mean insurance amount (NT$, SD)	14,623.96 (14,591.62)	12,966.04 (11,927.05)	15,523.42 (15,645.09)	15,010.48 (12,525.98)	17,004.99 (15,070.60)	15,534.37 (15,209.53)	16,269.37 (15,253.73)	0.0008
Socioeconomic status (%)								
< NT$20000	393 (37.64)	214 (40.30)	167 (46.39)	553 (47.80)	183 (52.14)	91 (42.72)	156 (47.27)	< 0.0001
NT$20000 ~ NT$39999	121 (11.59)	46 (8.66)	41 (11.39)	140 (12.10)	55 (15.67)	27 (12.68)	48 (14.55)	
≧NT$40000	64 (6.13)	19 (3.58)	28 (7.78)	48 (4.15)	32 (9.12)	21 (9.86)	27 (8.18)	
Union/association members	242 (23.18)	144 (27.12)	67 (18.61)	287 (24.81)	45 (12.82)	41 (19.25)	51 (15.45)	
Local government enrollees	224 (21.46)	108 (20.34)	57 (15.83)	129 (11.15)	36 (10.26)	33 (15.49)	48 (14.55)	
Urbanization of residence (%)								
Rural	119 (11.55)	71 (13.47)	30 (8.45)	151 (13.25)	27 (7.71)	18 (8.65)	18 (5.47)	0.0041
Sub-urban	361 (35.05)	196 (37.19)	138 (38.87)	445 (39.04)	98 (28.00)	66 (31.73)	114 (34.65)	
Urban	550 (53.40)	260 (49.34)	187 (52.68)	544 (47.72)	225 (64.29)	124 (59.62)	197 (59.88)	
Mean DCSI score (SD)	0.23 (0.65)	0.3 (0.69)	0.15 (0.45)	0.17 (0.47)	0.14 (0.44)	0.39 (0.73)	0.21 (0.53)	< 0.0001
Proportions of subgroups of DCSI (n, %)								
Low severity (DCSI = 0)	907 (86.88)	435 (81.77)	319 (88.61)	1013 (87.55)	312 (88.89)	156 (73.24)	277 (83.94)	0.3994
Moderate severity (DCSI = 1)	45 (4.31)	44 (8.27)	28 (7.78)	98 (8.47)	30 (8.55)	33 (15.49)	37 (11.21)	
High severity (DCSI≥2)	92 (8.81)	53 (9.96)	13 (3.61)	46 (3.98)	9 (2.56)	24 (11.27)	16 (4.85)	

Data are means ± SD or n (%).^*^Comparison across all seven groups. DCSI, diabetes complications severity index. NT$, New Taiwan dollar

**Table 2:** Odds ratios for predictors of trajectory membership related to trajectory of persist generalized group

	Persist irregular use	Generalized to irregular use	Irregular use to generalized	Generalized to specialized	Specialized to generalized	Persist specialized
	OR (95% CI)	OR (95% CI)	OR (95% CI)	OR (95% CI)	OR (95% CI)	OR (95% CI)
Gender (Ref: Female)						
Male	1.12 (0.94,1.34)	**1.52 (1.22,1.88)**	**1.29 (1.02,1.65)**	1.04 (0.81,1.33)	1.20 (0.89,1.62)	1.02 (0.79,1.30)
Age (Ref: 20–49)						
50–64	**0.65 (0.52,0.81)**	1.02 (0.76,1.37)	**0.61 (0.46,0.80)**	0.99 (0.74,1.31)	1.36 (0.90,2.07)	**1.43 (1.05,1.96)**
65–74	1.06 (0.83,1.36)	**2.09 (1.52,2.86)**	**0.70 (0.50,0.98)**	0.89 (0.62,1.27)	**2.12 (1.35,3.34)**	1.40 (0.97,2.03)
≧75	**3.34 (2.44,4.57)**	**5.19 (3.55,7.57)**	0.63 (0.37,1.08)	**0.31 (0.14,0.71)**	**3.89 (2.25,6.72)**	0.83 (0.44,1.56)
Socioeconomic status (Ref: <NT$20000)						
NT$20000 ~ NT$39999	1.22 (0.92,1.61)	0.85 (0.58,1.23)	0.95 (0.65,1.41)	1.19 (0.84,1.70)	1.17 (0.73,1.87)	1.22 (0.84,1.78)
≧NT$40000	**1.75 (1.17,2.61)**	0.96 (0.55,1.68)	**1.80 (1.09,2.97)**	**2.05 (1.27,3.33)**	**2.67 (1.52,4.72)**	**2.12 (1.28,3.53)**
Union/association members	1.06 (0.82,1.37)	0.99 (0.73,1.35)	0.96 (0.67,1.38)	0.67 (0.45,1.02)	0.87 (0.55,1.38)	0.87 (0.58,1.29)
Local government enrollees	**1.88 (1.44,2.46)**	**1.41 (1.02,1.93)**	**1.48 (1.02,2.14)**	0.94 (0.62,1.43)	1.22 (0.77,1.93)	1.36 (0.92,2.00)
Urbanization of residence (Ref: Rural)						
Sub-urban	1.05 (0.78,1.42)	1.00 (0.70,1.42)	1.48 (0.94,2.34)	1.01 (0.62,1.65)	1.18 (0.66,2.12)	**1.95 (1.12,3.38)**
Urban	1.34 (0.98,1.83)	1.15 (0.79,1.67)	1.59 (0.99,2.56)	**1.69 (1.03,2.76)**	1.79 (0.99,3.24)	**2.59 (1.48,4.55)**
DCSI of new-onset DM (Ref: Low severity, DCSI = 0)						
Moderate severity, DCSI = 1	**0.54 (0.37,0.78)**	1.03 (0.70,1.51)	0.96 (0.62,1.49)	1.01 (0.65,1.55)	**2.15 (1.39,3.32)**	1.35 (0.90,2.02)
High severity, DCSI≥2	**1.99 (1.36,2.89)**	**2.26 (1.48,3.44)**	0.93 (0.49,1.75)	0.68 (0.33,1.40)	**3.04 (1.79,5.15)**	1.25 (0.70,2.26)

Cells in boldface are statistically significant at *P* < 0.05. DCSI, diabetes complications severity index; OR, odds ratio. There were 2 patients who had missing data of gender and SES. One patient existed in the persist irregular use group, and the other exist in the generalized to irregular use group. The 2 patients were dropped from statistical analysis. There were 48 patients who had missing data of urbanization of residence. Fourteen patients existed in the persist irregular use group, 5 patients existed in the generalized to irregular use group, 5 patients existed in irregular use to generalized group, 17 patients existed in persist generalized group, 1 patient existed in generalized to specialized group, 5 patients existed in specialized to generalized group and 1 patient existed in persist specialized group. The 50 patients were dropped from statistical analysis

In terms of patients’ patterns of changing their provider levels, compared to patients who persistently visited generalized providers over time, patients aged 75 years or above were less likely to change their usual diabetes care source from generalized to specialized providers(adjusted OR = 0.31, 95% CI: 0.14,0.71) and more likely to follow the opposite trend (i.e. shifting their regular source of care from specialized care settings to generalized care settings) over time (adjusted OR = 3.89, 95% CI: 2.25,6.72). Considering baseline diabetes severity, the higher the severity of patients with diabetes, the more likely they were to change their usual diabetes care source from specialized to generalized providers and less likely to follow the opposite trend (i.e. shifting their regular source of care from generalized care settings to specialized care settings) over time. Considering SES, patients with diabetes with a monthly income of ≥NTD40,000 were significantly more likely either to change levels of their care setting or to persistently maintain regular visits to providers at specialized care settings. Similarly, urban residents were more likely to change their provider levels than persistently visiting generalized providers. Patients living in sub-urban or urban areas were more likely to be persistent regular uses of specialized providers over time.

## Discussion

This study is the first population-based assessment of long-term care-seeking behaviors of patients with type 2 diabetes in a setting with three interesting characteristics: a single-payer universal health insurance coverage, a fragmented delivery system, and the relative freedom of provider choice. We obtained a few critical findings. First, despite being covered by a comprehensive universal health insurance program over time, 26.19% of patients with type 2 diabetes had failed persistently to make regular visits to providers as recommended. More critically, an additional 13.34% of patients with type 2 diabetes who were initially regular seekers of diabetes care gradually lost their regular contacts with their providers over time. Only 9.03% of patients with type 2 diabetes changed from being irregular care-seekers to attending regular visits to generalized providers. Non-stable HbA1c level over time increased the risk of adverse diabetic complications and outcomes significantly [35]. Maintaining regular contacts with diabetes providers allows for regular monitoring of HbA1c levels and timely interventions for better patient outcomes. The findings suggest that the underutilization of recommended diabetes care remains a major challenge for diabetes care management in Taiwan, even after the NHI program substantially reduced the associated financial barriers.

Elderly patients, particularly those aged 75 years or more, and patients with high severity were at higher risks of being persistently or losing regular contacts with their providers. One plausible explanation is that potentially poorer accessibility to healthcare providers resulting from aging or disease severity may have limited the patient ability to maintain regular visits to providers. Disease-related knowledge, health literacy, disability, and cognitive function decline with aging [36, 37]. Another explanation may be that elderly patients are less motivated to regularly visit medical care providers because of lower expectations of benefits from medical services considering aging [38]. In contrast to studies with a shorter study period [37, 39], our findings provide novel evidence for the controversial long-term relationship between age and adherence to diabetes treatment.

Furthermore, patients with high baseline severity might have been those who were less attentive to their health or health behaviors. Therefore, it might have been difficult for them to adhere to regular diabetes care or management. Among all patients, those with moderate severity showed to have the highest likelihood of maintaining regular contacts with their providers in a long run. Compared with patients with least severity, the likelihood of diabetes-related symptoms was high among those with moderate severity, which might have compelled these patients to visit their providers regularly eventually. On the other hand, compared with patients with high severity, more favorable mobility capability among those with moderate severity might have reduced the physical barriers to regular diabetes care. DiMatteo et al. demonstrated that patients with moderate severity are more likely to be adherent than those in a healthy state, and patients with severe illness have an 11% higher risk of non-adherence than those in a healthy state [40]. Wagner and Ryan reported that maintaining treatment adherence is difficult in patients with severe chronic illness [41]. Patients with severe illness may doubt the efficacy of their treatments [42], and their expectations from healthcare providers may decrease as their condition worsens [43].

The relationship between SES and long-term care-seeking behaviors was interesting. Although the cost-sharing under the NHI program has been low or exempted for socioeconomically disadvantaged populations, the vulnerable patients such as the low income people were still not able to maintain their regular visits to providers. Patients with poor SES often have poor adherence to diabetes treatment [44]. Other non-medical costs related to care seeking and barriers to care shall be further investigated. However, we also found that patients with higher SES were less likely to maintain regular care-seeking behavior over time. One plausible explanation is that better ability to pay might have offered alternative care choices to patients with high SES. Garrow and Egede illustrated that in the United States, diabetes patients with a higher SES were more likely to use complementary or alternative medical services [45]. We only included visits under the NHI program and thus did not consider the alternative medical services sought and not reimbursed under the NHI program. Therefore, regular care-seeking behaviors of high SES may have been underestimated.

Second, approximately one-third of patients with diabetes had regularly and persistently received care from generalized care settings. Only 8.28% of patients had persistently sought care from specialized care setting. Patients with high SES, high severity, and residing in sub-urban and urban areas were more likely to visit specialized care settings such as medical centers and regional hospitals for diabetes care. Previous studies have also reported that higher SES patients with diabetes are more likely to receive specialist care, bypass closer generalized providers and directly visit specialized hospitals [46, 47]. The Medical Outcomes Study, a cross-sectional study conducted in the United States, reported that patients with severe diabetes were more frequently treated by specialists than by generalists, but the role of patient preference in this observation remains unknown [48]. Furthermore, Vanasseet al. demonstrated that patients with diabetes living in rural areas of Canada have poor accessibility to diabetes care and specialist [49]. A better ability to pay among patients with a higher SES, higher needs of more advanced treatments among patients with greater disease severity, and easier geographic proximity to specialized providers among urban and sub-urban patients might have led these patients to seek regular care from specialized providers over time, although the costs of seeking care from specialized providers were higher than those from generalized providers.

Third, approximately 27.50% of patients, who had persistently maintained regular visits to diabetes providers, changed the specialization level of their diabetes care providers over time. Higher SES patients and patients residing in urban areas were more likely to change to a different level of providers over time than being persistently visit generalized providers for diabetes care. These patients’ higher capability to pay, easier access to more information, and more favorable medical resource availability might have provided them more provider choices and facilitated changing the level of care. Notably, consistent with previous studies [46, 50], we found that elderly patients and those with higher severity were more likely to shift from specialized to generalized providers and less likely to follow the opposite trend. A shorter waiting time, more flexible clinic hours, and geographic proximity of generalized providers might have led elderly patients and those with higher severity, who tended to have poorer mobility, to this care seeking pattern.

Some limitations shall be noted. First, the presence of distinct developmental trajectories must be assumed a priori in a group-based trajectory approach [31]. A group-based trajectory approach cannot determine their presence. Second, although we have included few measures of SES and diseases severity, some inherited limitations of claims data including the lack of laboratory test results and income may lead to possible confounding and misclassification bias. The NHI Research Database lacks information on health care services that are not covered by the NHI program, such as complementary and alternative medicine. Patients with diabetes who sought diabetes care at their own expense might have been considered as not maintaining regular contacts with the mainstream diabetes care providers in our study. More detailed clinical information and socioeconomic status information may help to reduce such biases. Third, the NHI claims data lack detailed clinical data or information on the reasons why people changed their care seeking behaviors. Due to data limitations, we are not able to further explore the details of care seeking decision processes. Future research with more detailed information can help to contribute in this regard. Furthermore, due to data limitation, only major specialty categorization information such as general practice, surgery, internal medicine, pediatrics, and obstetrics/gynecology is available in the claims data. We are unable to include physician’s sub-specialty in our analysis. Future research with more detailed information on physician’s specialty can help to contribute in this regard.

## Conclusions

The most alarming long-term health care pattern observed in this study was that, despite the existence of the comprehensive universal health insurance coverage by the NHI program in Taiwan, almost 50% of patients with type 2 diabetes still could not maintain regular visits to diabetes providers as recommended over time. Age, SES, residential location, and disease severity were significant predictors of long-term health care-seeking behaviors of these patients, including both regularity of care seeking and specialization level of their providers. Our findings provide policy makers and researchers a more comprehensive understanding of the long-term trajectories of care-seeking behaviors of patients with chronic conditions, such as diabetes. More effective strategies for improving regularity in care seeking and strengthening functions of diabetes providers in chronic care delivery may be developed.

## Data Availability

Both the NHIRD (http://nhird.nhri.org.tw/en/index.htm) and LHID databases (http://nhird.nhri.org.tw/en/Data_Subsets.html) are regulated and managed by the Ministry of Health and Welfare. According to the official regulations, both databases (NHIRD and LHID) are only open to academic personnel in Taiwan for research purposes. Researchers from other countries can gain access to the data sets for research purposes by collaborating with researchers in Taiwan and submitting an official data application to the Ministry of Health and Welfare. Applicants must follow the Computer-Processed Personal Data Protection Law and the regulations of the Ministry of Health and Welfare. All applications need to obtain an IRB approval before their submission for data application. The data that support the findings of this study are available from the Ministry of Health and Welfare (https://dep.mohw.gov.tw/DOS/np-2497-113.html), but restrictions apply to the availability of these data, which were used under license for the current study, and so are not publicly available. Data are however available from the authors upon reasonable request and with permission of the Ministry of Health and Welfare.

## Abbreviations

APP: Average posterior probability
BIC: Bayesian information criterion value
CI: Confidence interval
DCSI: Diabetes complications severity index
DM: Diabetes mellitus
HbA1c: Glycated hemoglobin
ICD-9-CM: The International Classification of Diseases, Ninth Revision, Clinical Modification
LHID: Longitudinal Health Insurance Database
NHI: National Health Insurance
NHIRD: National Health Insurance Research Database
NTD: New Taiwan dollars
OR: Odds ratio
SES: Socioeconomic status
USD: United States dollars
